# Association of Predicted Lean Body Mass and Fat Mass With Incident Diabetic Nephropathy in Participants With Type 2 Diabetes Mellitus: A Post Hoc Analysis of ACCORD Trial

**DOI:** 10.3389/fendo.2021.719666

**Published:** 2021-10-27

**Authors:** Daniel Nyarko Hukportie, Fu-Rong Li, Rui Zhou, Meng-Chen Zou, Xiao-Xiang Wu, Xian-Bo Wu

**Affiliations:** ^1^ Department of Epidemiology, School of Public Health, Southern Medical University (Guangdong Provincial Key Laboratory of Tropical Disease Research), Guangzhou, China; ^2^ School of Medicine, Southern University of Science and Technology, Shenzhen, China; ^3^ Department of Endocrinology and Metabolism, Nanfang Hospital, Southern Medical University, Guangzhou, China; ^4^ Department of General Surgery, 157^th^Hospital, General Hospital of Guangzhou Military Command, Guangzhou, China

**Keywords:** obesity, lean body mass, fat mass, diabetes, diabetic nephropathy

## Abstract

**Background:**

Lean body mass (LBM) and fat mass (FM) have been shown to have different associations with several chronic diseases but little is known about the sex-specific association of LBM and FM with diabetic nephropathy (DN) risk among participants with diabetes.

**Methods:**

Participants from the Action to Control Cardiovascular Risk in Diabetes (ACCORD) study was used in a *post hoc* analysis to examine the association of predicted LBM index (LBMI) and FM index (FMI) with incident DN risk (defined as a composite outcome of three types of predefined DN). Because of sex differences in body composition, analyses were conducted separately using sex-specific quartiles of predicted LBMI and FMI.

**Results:**

Of the 9,022 participants with type 2 diabetes (5,575 men and 3,447 women) included in this study, 5,374 individuals developed DN (3,396 in men and 1,978 in women). Higher quartiles of LBMI were associated with a reduced risk of DN while higher quartiles of FMI were associated with an increased higher risk of DN among men but not women. Compared with the lowest quartile, the fully adjusted hazard ratios (HRs) and 95% confidence intervals (CIs)for the highest quartile of predicted LBMI and FMI were respectively 0.83 (95% CI 1.71 – 0.96) and 1.23 (95% CI 1.06-1.43) among men; and 0.92 (95% CI 0.63 – 1.33) and 1.14 (95% CI 0.79 – 1.63) among women.

**Conclusions:**

Among participants with diabetes, predicted LBMI was inversely associated with risk of DN while predicted FMI was positively associated with an increased risk of incident DN among men but not women. Trial registration: ClinicalTrials.gov., no. NCT00000620.

## Introduction

Globally, the incidence and prevalence of diabetes mellitus have been on the increase and have since been recognized as one of the rapidly growing global health emergencies of the 21^st^ century (1, 2). According to estimates from the International Diabetes Federation, there were about 463 million people living with diabetes globally in 2019, a figure that has been projected to reach about 700 million people by 2045 coupled with a potential increase in diabetic complications (2). One of the associated complications for diabetes is diabetic nephropathy (DN) – a microvascular complication characterized by albuminuria and progressive loss of kidney function (3); and is also considered to be the leading cause of end-stage renal disease (ESRD) in the United States (4) and across the globe (5, 6).

Several factors including obesity have been identified to be associated with DN risk among individuals with diabetes (7). Indeed, a number of studies have reported a positive relationship between renal disease and being overweight among both men and women with or without diabetes (8–12). However, other studies found a sex-specific association between obesity and renal disease risk (13). For example, in a study of patients with diabetes managed in a care setting, Yu et al. found diabetic kidney disease to be more prevalent among men compared with women although women were more likely to be overweight than men (14); which is similar to the findings of Shankar et al. who also found higher body mass index (BMI) to be associated with an increased risk of renal disease in men but not in women (15). On the other hand, Noh et al. found that higher levels of BMI were linked to a higher risk of albuminuria in women but not men in a population of Korean non-diabetic adults (13).

Although BMI is a widely used measure of obesity, some studies have found that BMI is not without deficiency in differentiating between lean body mass (LBM) and fat mass (FM) (16, 17). Since body composition may vary between individuals and sexes, people with the same BMI level are likely to have different body compositions and also a different risk of morbidities (18). Having indices that could distinguish between LBM and FM is essential as some studies suggest a somewhat protective effect of LBM while a detrimental effect of FM on health outcomes (19, 20). However, studies that require the use of data on a direct measure of LBM and FM comes at high costs and more so when it has to do with a large sample size because it involves the use of sophisticated technologies such as dual energy radiographic absorptiometry or imaging technologies (20). This poses great challenge in studying the influence of LBM and FM on health risks in large cohort studies, hence until now, little is known about the effect of LBM and FM on DN risk among patients with diabetes. To address the limitation imposed by cost and sophisticated measuring of direct LBM and FM, several anthropometric equations taken into consideration in relation between body measurement and body composition have been developed (21). One of such equation that has been previously used among the Action to Control Cardiovascular Risk in Diabetes (ACCORD) study by Xing et al. is the validated equation developed by Lee et al. using a large cross-sectional sample from the National Health and Nutrition Examination Survey (NHANES) (16, 17).

The prediction equations mentioned above permit studies of LBM or FM without dual-energy x-ray absorptiometry or imaging technologies. In this context, we conducted a *post hoc* analysis to investigate the association of predicted LBM and FM with incident DN using data from the ACCORD study, which was a randomized, multicenter, double 2 × 2 factorial trial in 10,251 patients with type 2 diabetes mellitus (T2DM) (22).

## Methods

### Study Participants and Data Collection

ACCORD was a randomized clinical trial of 10,251 participants with T2DM who were followed with the objective of assessing the health effects of intensive glycemic, lipid, and blood pressure (BP) control as against standard control (23, 24). The design and main results of the ACCORD study have been published previously (24). Briefly, accord had three study arms (1) glycaemia trial (glycated hemoglobin [HbA1c] <6.0% vs 7.0% < HbA1c <7.9%); (2) lipid trial (fenofibrate vs placebo) and (3) BP trial (systolic BP <120mmHg vs systolic BP <140mmHg) with all participants involved in the glycaemia trial (25). Recruitment of participants into the study began in January 2001 through to October 2005 from 77 clinical sites across Northern America (i.e. US and Canada) (26). Ethical approval for the ACCORD study was granted by institutional review boards of each clinical site and written informed consent was obtained from all recruited participants (trial registration: ClinicalTrials.gov., no. NCT00000620).

**Figure 1:**
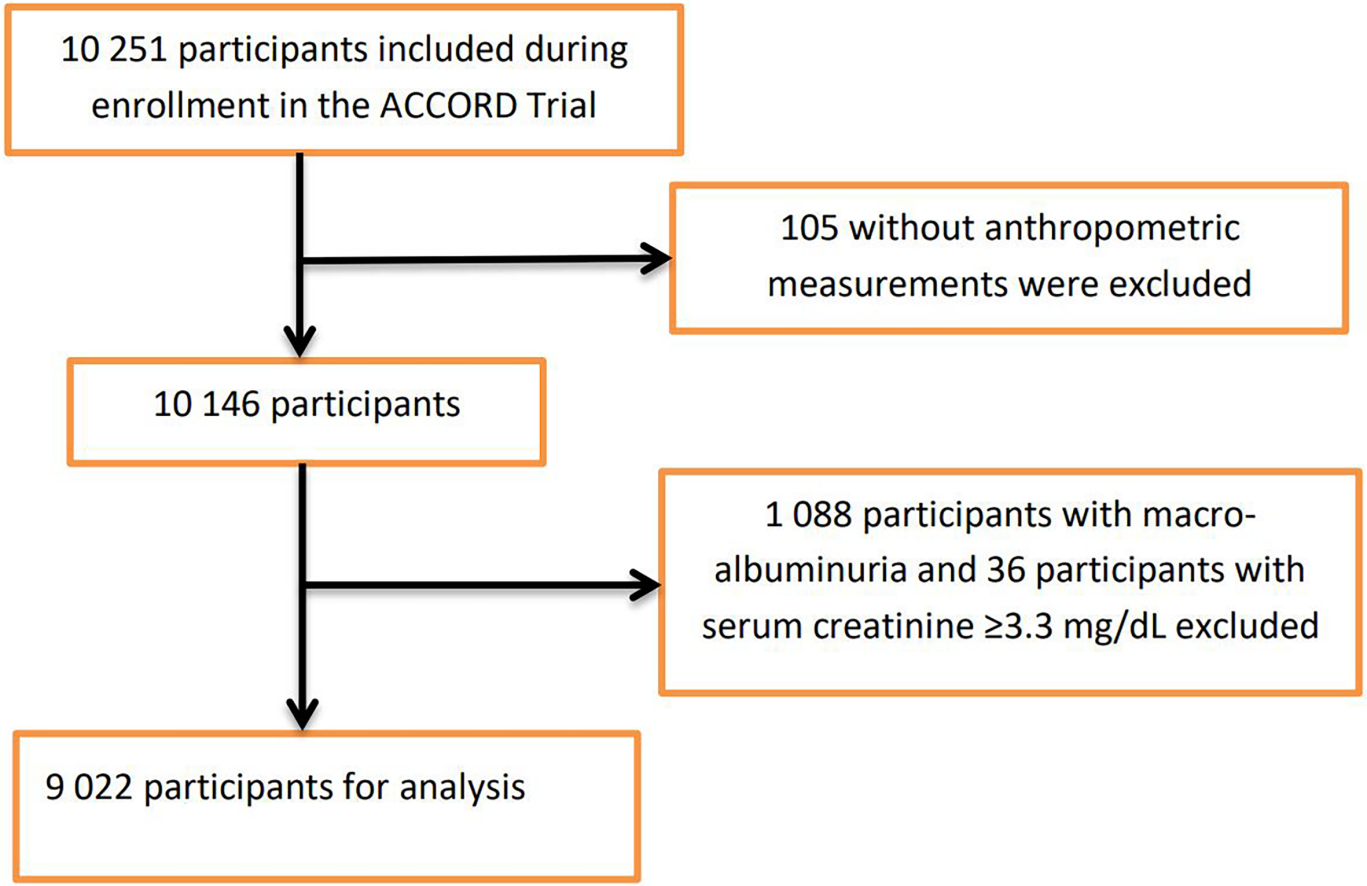
Flowchart for participant enrollment.

For this *post hoc* study, participants were excluded if they had missing anthropometric measurements at baseline or with prevalent baseline nephropathic conditions that formed part of the predefined ACCORD nephropathic incident event: macro-albuminuria ≥300mg/g and serum creatinine clearance ≥3.3mg/dL (**Supplementary Table 1**). **Figure 1** shows the flowchart of the selection of the analytic sample.

### Exposure Variables

Predicted LBM index (LBMI) and predicted FM index (FMI) were computed using prediction equations developed by Lee et al. using participants from the NHANES survey, which included 7,531 men and 6,534 women who were examined using dual-energy radiographic examination (16). They used multivariable linear regression to estimate lean body mass and fat mass measured by dual-energy radiography as dependent variables with the following anthropometric measures as predictor variables: age, sex, race, height (cm), weight (kg), and waist circumference (cm) (**Supplementary Table 2**) (16). The estimated values for lean body mass and fat mass from these equations were respectively divided by height in meters to generate the predicted LBMI and predicted FMI since lean body mass and fat mass have been shown to correlate with height (17).

### Study Outcome

The study outcome was an incidence of DN defined as (1) doubling of serum creatinine or >20 mL/min/1.73m^2^ decrease in estimated glomerular filtration rate (eGFR); (2) urine albumin/creatinine ratio (uacr) ≥300 mg/g (3) renal failure or ESRD (dialysis) or serum creatinine (SCr) >3.3mg/dL in absence of an acute reversible cause (**Supplementary Table 1**). A composite score of these events was used as the outcome. Participants who experience any one of these events specified by the ACCORD study were considered to have experienced the composite outcome.

### Statistical Analysis

Participants’ characteristics were described using mean ± standard deviation (SD) or median (25th and 75th percentiles) for continuous variables, depending on whether the data distribution was normal (assessed by the Shapiro-Wilk test); categorical variables were described by frequencies and percentage. Analysis of variance, Mann-Whitney U test, or χ2 was used to determine differences between predefined groups.

Since body composition varies between men and women, and so is the incidence and health consequence associated with these sex-specific differences (18), the analysis was sex-stratified. Predicted LBMI and predicted FMI were divided into sex-specific quartiles. Cox proportional hazards regression models with time-dependent covariates were used to estimate hazard ratios (HRs) with 95% confidence intervals (CIs) for risk of DN using the lowest sex-specific quartile of predicted LBMI and predicted FMI as the reference group. The participants were followed up from baseline until the time of development of the composite DN outcome, time of death, loss to follow-up, or the end of the follow-up, whichever occurred first.

Predicted LBMI and predicted FMI were mutually adjusted; three models were used to examine the associations. Model 1 adjusted for age (continuous), race (Black, Hispanic, Other and White), glycemia (intensive and standard), BP vs lipid (BP standard, BP intensive, Lipid Placebo, and Lipid Fibrate), and diabetes duration (continuous); Model 2 adjusted for model 1 variables plus alcohol consumption (yes and no), and cigarette (yes and no); Model 3 adjusted for model 2 variables plus hypertension history (yes and no), stroke history (yes and no) and cardiovascular disease history (yes and no). Tests for linear trends were carried out by entering the median value of each category of predicted LBMI or predicted FMI as a continuous variable in the models. Restricted cubic spline analysis was used to visualize the relation between predicted LBMI and predicted FMI with incident DN.

Further exploration was carried out to examine possible interactions between each 2kg/m^2^ increase in predicted LBMI/FMI and the following baseline characteristics of the participants with respect to incident DN: age (≤60 or > 60 years), diabetes duration (≤9 or >9 years), race (white or non-white) hypertension history, cardiovascular disease history, cigarette smoking history, glycaemia therapy (intensive or standard) and BMI (<25kg/m^2^ or ≥25kg/m^2^). The statistical significance of the interactions was assessed by adding a multiplicative term to the Cox models.

Sensitivity analyses were also performed by excluding participants with any serious adverse event (attributed to study medication and not related to hypoglycemia) that occurred in the first 2 years of follow-up, or by further adjustments for insulin use at baseline. All statistical analyses were 2-sided, and we considered a *p-*value of < 0.05 to be statistically significant. All analyses were performed using Stata (version 16 MP; StataCorp, College Station, Texas) and Microsoft Excel.

## Results

**Table 1** showed the baseline characteristics of the analytic sample of 9,022 participants included in this study. Compared with men, women tended to be younger, non-smokers, non-drinkers, less likely to have a history of cardiovascular disease, and have higher predicted FMI and HbA1c levels but lower levels of predicted LBMI, uacr, SCr and lower eGFR.

**Table 1:** Baseline characteristics of included participants by sex.

Characteristic	Men	Women	*P* value
No. of participants	5,575 (61.79)	3,447 (38.21)	
Predicted LBMI, kg/m^2^	20.68 (19.04-22.66)	17.52 (16.02-18.98)	0.0001
Predicted FMI, kg/m^2^	9.76 (8.06-11.75)	15.02 (12.41-17.72)	0.0001
Age, y	62.2 (57.8 – 67.6)	61.7 (57.6 – 66.4)	0.0002
Glycaemia therapy			
Intensive	2,785 (49.96)	1,737 (50.39)	0.687
Standard	2,790 (50.04)	1,710 (49.61)	
Race			
White	3,765 (67.53)	1,913 (55.50)	<0.001
Black	862 (15.46)	837 (24.28)	
Hispanic	333 (5.97)	304 (8.82)	
Other	615 (11.03)	393 (11.40)	
Diabetes duration, y	9 (5 - 15)	9 (5 - 15)	0.6217
Hypertension	4,994 (89.58)	3,109 (90.19)	0.347
History of CVD	2,265 (40.63)	866 (25.12)	<0.001
Stroke	332 (5.96)	197 (5.72)	0.637
Smoking	833 (14.94)	396 (11.49)	<0.001
Alcohol use	1,777 (31.87)	420 (12.18)	<0.001
Arm of the trial			
Lipid fibrate	1,687 (30.26)	726 (21.06)	<0.001
Lipid placebo	1,689 (30.30)	721 (20.92)	
Intensive BP	1,106 (19.84)	1,008 (29.24)	
Standard BP	1,093 (19.61)	992 (28.78)	
Glycosylated hemoglobin, %	8.26 (1.03)	8.33 (1.07)	0.001
Insulin use	1781 (31.9)	1262 (36.6)	
Serum creatinine, mg/dL	1 (0.8 – 1.1)	0.8 (0.7 – 0.9)	0.0001
UACR, mg/g	14 (7 - 38)	12 (7 - 27)	0.0001
eGFR, mL/min/1.73m^2^	89.9 (77.9-104.1)	89.5 (74.2-107.7)	0.0138

Data are shown as medians (interquartile range) for continuous variables and as frequencies and percentages for categorical variables.

BP, blood pressure; LBMI, lean body mass index; FMI, fat mass index.

Over 24,659 person-years of follow-up, 5,374 participants (3,396 men and 1,978 women) developed the DN composite outcome (2.3 per 10 person-years in men; 2.1 per 10 person-years in women). **Tables 2**, **3** showed the association of predicted LBMI and predicted FMI with the risk of incident DN among men and women respectively. Among men, higher levels of predicted LBMI were significantly associated with a lower risk of incident DN, with a fully-adjusted HR (95% CIs) of 0.83 (0.71 – 0.96) for the highest quartile compared with the lowest quartile. On the contrary, higher levels of predicted FMI were inversely associated with the risk of incident DN: the fully-adjusted HR (95% CIs) was 1.23 (1.06 – 1.43) for the highest quartile comparing with the lowest quartile. Significant linear trends were found for both predicted LBMI and predicted FMI. Although women also tended to show reduced and increased risk of DN with higher quartiles of predicted LBMI and predicted FMI, the associations did not reach statistical significance. The corresponding fully-adjusted HRs (95% CIs) were 0.92 (0.63 – 1.33) for predicted LBMI and 1.14 (0.79 – 1.63) for predicted FMI among women (**Table 3**).

**Table 2:** Hazard ratios for incident DN, by predicted LBMI and predicted FMI among men with diabetes.

Men (n=5,575)	No. of events	Incidence Density (Per 10 Person Years)	HR (95% CI)		
			Model 1	Model 2	Model 3
LBMI quartile					
1	829	2.17	Ref.	Ref.	Ref.
2	847	2.21	0.97 (0.87 – 1.08)	0.97 (0.87 – 1.09)	0.97 (0.87 – 1.08)
3	873	2.36	0.93 (0.81 – 1.05)	0.93 (0.82 – 1.06)	0.93 (0.82 – 1.06)
4	847	2.29	0.82 (0.71 – 0.96)*	0.83 (0.71 – 0.97)*	0.83 (0.71 – 0.96)*
*p* value of trend			0.011	0.015	0.014
FMI quartiles					
1	812	2.10	Ref.	Ref.	Ref.
2	827	2.14	1.00 (0.90 – 1.11)	1.00 (0.90 – 1.11)	0.99 (0.89 – 1.11)
3	880	2.36	1.12 (1.99 – 1.27)	1.12 (0.99 – 1.27)	1.11 (0.98 – 1.26)
4	877	2.46	1.25 (1.08 – 1.45)**	1.25 (1.08 – 1.44)**	1.23 (1.06 – 1.43)**
*p* value of trend			0.001	0.001	0.002
LBMI/2kg/m^2^			0.95 (0.91 – 0.99)*	0.95 (0.91 – 0.99)*	0.95 (0.91 – 0.99)*
FMI/2kg/m^2^			1.07 (1.03 – 1.12)**	1.07 (1.03 – 1.12)**	1.07 (1.02 – 1.11)**

LBMI, lean body mass index; FMI, fat mass index; CI, confidence interval; HR, hazard ratio; Ref., reference.

Model 1 adjusted for age (continuous), race (Black, Hispanic, Other and White), glycaemia (intensive and standard), BP vs lipid (BP standard, BP intensive, Lipid Placebo, and Lipid Fibrate), and diabetes duration (continuous).

Model 2 adjusted for model 1 variables plus alcohol (yes and no), and cigarette (yes and no).

Model 3 adjusted for model 2 variables plus hypertension history (yes and no), stroke history (yes and no), and cardiovascular disease history (yes and no).

p-value notation: *p < 0.05. **p < 0.01. ***p < 0.001.

*Both predicted LBMI and predicted FMI were mutually adjusted for each other.

**Table 3:** Hazard ratios for incident DN, by predicted LBMI and predicted FMI among women with diabetes.

Women (n=3,447)	No. of events	Incidence Density (Per 10 Person Years)	HR (95% CI)		
			Model 1	Model 2	Model 3
LBMI quartiles					
1	481	1.94	Ref.	Ref.	Ref.
2	468	1.89	0.98 (0.79 – 1.20)	0.97 (0.79 – 1.20)	0.97 (0.78 – 1.19)
3	513	2.20	0.98 (0.74 – 1.32)	0.98 (0.73 – 1.31)	0.97 (0.72 – 1.30)
4	516	2.22	0.94 (0.65 – 1.36)	0.93 (0.64 – 1.35)	0.92 (0.63 – 1.33)
*p* value of trend			0.865	0.838	0.786
FMI quartiles					
1	485	1.98	Ref.	Ref.	Ref.
2	465	1.84	0.95 (0.77 – 1.16)	0.94 (0.77 – 1.16)	0.95 (0.77 – 1.16)
3	512	2.22	1.11 (0.84 – 1.48)	1.10 (0.83 – 1.46)	1.12 (0.84 – 1.48)
4	516	2.21	1.14 (0.79 – 1.63)	1.13 (0.78 – 1.62)	1.14 (0.79 – 1.63)
*p* value of trend			0.448	0.483	0.457
LBMI/2kg/m^2^			0.93 (0.81 – 1.06)	0.92 (0.81 – 1.05)	0.92 (0.80 – 1.05)
FMI/2kg/m^2^			1.00 (0.93 – 1.07)	1.00 (0.93 – 1.07)	1.00 (0.93 – 1.07)

LBMI, lean body mass index; FMI, fat mass index; CI, confidence interval; HR, hazard ratio; Ref., reference.

Model 1 adjusted for age (continuous), race (Black, Hispanic, Other and White), glycaemia (intensive and standard), BP vs lipid (BP standard, BP intensive, Lipid Placebo, and Lipid Fibrate), and diabetes duration (continuous).

Model 2 adjusted for model 1 variables plus alcohol (yes and no), and cigarette (yes and no).

Model 3 adjusted for model 2 variables plus hypertension history (yes and no), stroke history (yes and no), and cardiovascular disease history (yes and no).

p-value notation: *p < 0.05. **p < 0.01. ***p < 0.001.

*Both predicted LBMI and predicted FMI were mutually adjusted for each other.

**Figure 2:**
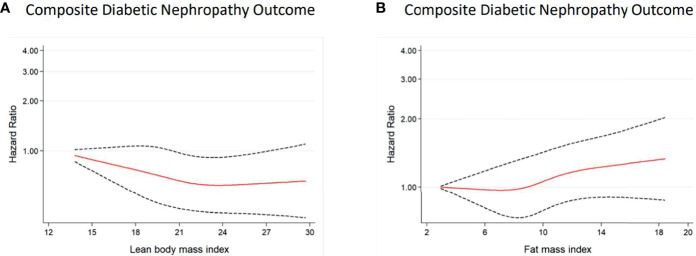
Multivariable-adjusted hazard ratios of incident DN for predicted LBMI **(A)** and predicted FMI **(B)** among *men* with diabetes. Curves represent adjusted hazard ratios for DN based on restricted cubic splines with knots placed at 5th, 35th, 50th, 65th, and 95th centiles. Models were adjusted for baseline age, race, glycaemia, BP vs lipid, diabetes duration, alcohol, cigarette, hypertension history, stroke history, and CVD history. Predicted LBMI and predicted FMI were mutually adjusted. The solid red line represents the hazard ratio, and the dashed black lines represent the 95% confidence intervals for the association between LBMI or FMI and DN composite outcome.

**Figure 3:**
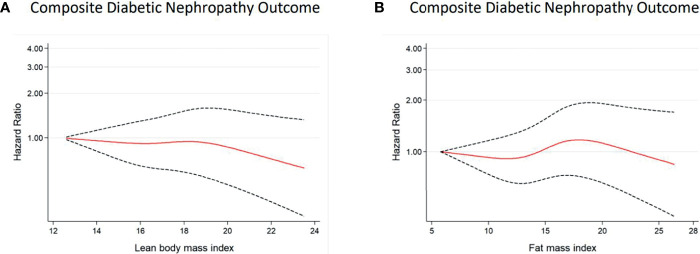
Multivariable-adjusted hazard ratios of incident DN for predicted LBMI **(A)** and predicted FMI **(B)** among *women* with diabetes. Curves represent adjusted hazard ratios for DN based on restricted cubic splines with knots placed at 5th, 35th, 50th, 65th, and 95th centiles. Models were adjusted for baseline age, race, glycaemia, BP vs lipid, diabetes duration, alcohol, cigarette, hypertension history, stroke history, and CVD history. Predicted LBMI and predicted FMI were mutually adjusted. The solid red line represents the hazard ratio, and the dashed black lines represent the 95% confidence intervals for the association between LBMI or FMI and DN composite outcome.

Similar patterns of relationships were observed in the restricted cubic spline analyses (**Figures 2**, **3**). A linear association of both predicted LBMI and FMI with DN was observed in men. The risk of DN declined as predicted LBMI increased until approximately 23kg/m^2^ when the trend plateaued; whereas increasing predicted FMI also showed detrimental association with incident DN but only after reaching about 8kg/m^2^ (**Figure 2**). For women, however, no clear trends were noted for both predicted LBMI and FMI (**Figure 3**).

Among men, each 2kg/m^2^ increase in predicted LBMI was associated with reduced risk of incident DN whiles each 2kg/m^2^ increase in predicted FMI was associated with an elevated risk of incident DN (**Table 2**) but this relationship was null among women (**Table 3**). The results were not materially changed after excluding those who had serious adverse effects in the first 18 months or additionally adjusting for baseline insulin use (**Supplementary Tables 3**, **4**). Subgroup analyses of the HRs of DN for each 2kg/m^2^ increase in the predicted LBMI and predicted FMI are shown in **Supplementary Figures 1**–**4**. Generally, there were no significant interactions between various subgroups.

## Discussion

In this *post hoc* study of 9,022 participants with T2DM, we found a sex-specific association of predicted LBMI and predicted FMI with incident DN. Specifically, higher levels of predicted LBMI were associated with a lower risk of incident DN among men with diabetes, while higher levels of predicted FMI were linked to an increased risk of incident DN. However, null associations were found among women with diabetes. Results of subgroup analysis and sensitivity analysis generally confirmed the above-mentioned observations.

High levels of FM have been demonstrated to be detrimental to health in some studies among both individuals with diabetes and without diabetes. For example, Xing et al. found a high level of predicted FM to be associated with an increased risk of CVD among 10,251 patients with T2DM (17). Similarly, Hanai et al. found increased visceral fat to be independently associated with microalbuminuria (an indicator of renal function compromise), using a hospital-based study of 208 adult Japanese with T2DM (27). Furthermore, Lee et al (20), and, Medina‐Inojosa et al. (28) in their respective study samples of 38,006 adult men and 717 patients with a history of cardiac artery disease (CAD) found a higher level of FM to be associated with CVD risks. Our results for men with diabetes were consistent with these findings suggesting that higher FM may pose an increased risk of morbidity including renal function decline. Although the exact mechanism is not entirely understood (29), however, it has been suggested that excess adipose tissue may affect the incidence and progression of kidney disease through several pathways including altering renal blood circulation (30), the release of inflammatory mediators such as interleukin-6 or tumor necrosis factor-α (31, 32), and worsening of insulin resistance, which may be associated with renal damage (33). Additionally, excess fat mass surrounding kidney tissues can also increase glomerular pressure which may, in turn, impair renal function (34).

On the other hand, our study also found higher levels of LBMI among men to be associated with a lower risk of DN which is consistent with other studies reporting a protective effect of higher LBM (35–38). For example, Spahillari et al. in a study of 1,335 elderly participants with a mean age of 76.2 ± 4.8 years, found greater lean mass to be associated with lower cardiovascular and total mortality (39). Similarly, Medina‐Inojosa et al. in the study of participants with a history of CAD, found lean mass to be associated with reduced risk of a major adverse cardiovascular event (28). Furthermore, Hong et al. found skeletal muscle mass index to be inversely associated with risk of T2DM in a cohort of 203,767 men and women with a median age of 39.1 years at baseline (40). The possible mechanism underlying the health benefit of high LBM includes its improvement in insulin sensitivity and maintaining healthy blood glucose levels which could subsequently prevent stress on the kidneys (41, 42). Furthermore, a high level of LBM is considered a vital source of protein reserve necessary in the defense of the body against adverse clinical outcomes (43).

Our results however were contrary to other studies investigating the relationship of obesity with declining renal function in both men and women. For Example, Oh et al. found higher categories of WC, a surrogate of obesity, to be significantly associated with a higher risk of renal function deterioration among both men and women, but surprisingly, they did not find body fat mass and body fat percent (estimated by bioelectrical impedance analysis) to be significantly associated kidney function decline in a prospective cohort study of 1,520 Korean participants (34). Also, Noh et al. found higher quartiles of WC and BMI among women to be associated with a higher risk of albuminuria, an observation which was absent in male participants (13). In our study, however, neither predicted FM nor predicted LBM yielded any significant association with DN among women with diabetes although women generally had higher FMI and lower LBMI than men. The exact reasons for these observed sex-specific differences in our study are not clear. Some studies have demonstrated that men are at greater risk of developing renal disease, and also tended to develop renal disease earlier in life than women (15, 44–48). Potential biological mechanisms may include the influence of sex hormones which have been implicated as modifying the pathway of body mass and renal disease. For example, estrogen has been shown to stimulate renal nitric oxide production and to have antioxidant characteristics (49) whereas androgens may increase arterial pressure by activating the renin-angiotensin system and subsequently leading to hyperfiltration, glomerular hypertension, and sclerosis (50, 51). Besides this possible explanation, it has also been suggested that male sex hormones may be involved in a mechanism in which androgens may contribute to deleterious effects on renal cells by interacting with other factors that may occur in several conditions including obesity (15, 52). Further investigation is needed to explore the gender-related differences in the associations between lean body mass and fat mass with DN.

The strengths of our study include the relatively large sample size, comprehensive clinical and biological characterization of participants, and prospective data collection. Meanwhile, our study has some limitations. Firstly, the ACCORD study did not directly collect data on fat mass or lean body mass; hence the use of predicted LBMI and predicted FMI might not be a perfect indicator of true lean body mass and fat mass. However, the prediction equations developed by Lee et al. using data from the NHANES survey exhibited high predictive ability of the anthropometric equation (with a correlation of >0.90) between fat mass or lean body mass and the direct dual-energy radiographic examination (16). Secondly, our study lacks the ability to determine causality because of its observational study design. Thirdly, the generalization of these findings to other populations may be limited since all study participants were from northern America and there may be variability in body composition across different populations.

In conclusion, our study indicates that among participants with diabetes, predicted LBMI was inversely associated with risk of DN while predicted FMI was positively associated with an increased risk of incident DN among men but not women. Men with T2DM should have sufficient LBM and less FM to reduce the risk of DN.

## Data Availability Statement

Publicly available datasets were analyzed in this study. This data can be found here: Biologic Specimen and Data Repository (https://biolincc.nhlbi.nih.gov/studies/accord/).

## Ethics Statement

The studies involving human participants were reviewed and approved by The Protocol Review Committee, appointed by the National Heart, Lung, and Blood Institute (NHLBI). Each clinical site’s local institutional review board also approved the study. The patients/participants provided their written informed consent to participate in this study.

## Author Contributions

DH and F-RL conceived, analyzed the data, and wrote the manuscript. RZ, M-CZ, X-XW, and X-BW contributed to the discussion and reviewed/edited the manuscript. All authors contributed to the article and approved the submitted version.

## Funding

This study was supported by the National Natural Science Foundation of China (82173607), the Guangdong Basic and Applied Basic Research Foundation (2021A1515011684), Open Project of the Guangdong Provincial Key Laboratory of Tropical Disease Research (2020B1212060042) and Guangzhou Science and Technology Project (202102080597).

## Conflict of Interest

The authors declare that the research was conducted in the absence of any commercial or financial relationships that could be construed as a potential conflict of interest.

## Publisher’s Note

All claims expressed in this article are solely those of the authors and do not necessarily represent those of their affiliated organizations, or those of the publisher, the editors and the reviewers. Any product that may be evaluated in this article, or claim that may be made by its manufacturer, is not guaranteed or endorsed by the publisher.
