# Supplementary material for: Association of Predicted Lean Body Mass and Fat Mass With Incident Diabetic Nephropathy in Participants With Type 2 Diabetes Mellitus: A Post Hoc Analysis of ACCORD Trial
Source: Front Endocrinol (Lausanne). 2021 Oct 27;12:719666. doi: 10.3389/fendo.2021.719666 (PMC8578879; doi:10.3389/fendo.2021.719666)
Supplement: Supplementary file 1 [file DataSheet_1.docx]

Supplementary Material

**Supplementary Table 1** Definition of DN and their frequency of assessment

| **Outcome** | **Definition** | **Assessment Frequency** |
| --- | --- | --- |
| Nephro-1 | SCr doubling or >20 mL/min decrease in eGFR | Every 4 month |
| Nephro-2 | Development of macro-albuminuria (uacr>=300mg/g) | Annually |
| Nephro-3 | Renal failure OR ESRD (dialysis) OR SCr>3.3 mg/dL in absence of an acute reversible cause | Every 4 month |
|  |  |  |

Abbreviations: DN = Diabetic Nephropathy. SCr = Serum creatinine. eGFR = Estimated glomerular filtration rate. uacr = Urinary albumin/creatinine ratio. ESRD = End stage renal disease

**Supplementary Table 2** Anthropometric prediction equations for LBM and FM

| **lean body mass** | **Equation** | **R^2^** | **SEE(kg)** |
| --- | --- | --- | --- |
| **Men** | 19.363 + 0.001*age (years) + 0.064*height (cm) + 0.756*weight (kg) –0.366*waist (cm) – 0.066*Mexican + 0.231*Hispanic + 0.432*Black – 1.007* Other ethnicity | 0.91 | 2.55 |
| **Women** | -10.683–0.039*age(years)+0.186*height(cm)+0.383*weight(kg)-0.043*waist(cm)0.359*Mexican–0.059*Hispanic+1.085*Black -0.34 *Other ethnicity | 0.85 | 2.38 |
| **Fat mass** |  |  |  |
| **Men** | -18.592–0.009*age(years)–0.080*height(cm)+0.226*weight(kg)+ 0.387*waist(cm)+0.080*Mexican–0.188*Hispanic–0.483*Black + 1.050*Other ethnicity | 0.90 | 2.60 |
| **Women** | 11.817+0.041*age(years)–0.199*height(cm)+0.610*weight(kg)+ 0.044*waist(cm)+0.388*Mexican+0.073*Hispanic–1.187*Black+0.325*Other ethnicity | 0.93 | 2.44 |

Reference: Lee, D.H., et al., *Development and validation of anthropometric prediction equations for lean body mass, fat mass and percent fat in adults using the National Health and Nutrition Examination Survey (NHANES) 1999-2006.* Br J Nutr, 2017. **118**(10): p. 858-866.

**Supplementary Table 3** Sensitivity analysis of LBMI and FMI in relation to DN among men and women by further adjusting for insulin at baseline

|  | | **HR (95% CI)** | | **HR (95% CI)** |
| --- | --- | --- | --- | --- |
| **Men** | |  | |  |
| Quartiles | | Predicted LBMI | | Predicted FMI |
| 1 | | Ref. | | Ref. |
| 2 | | 0.97 (0.87 – 1.08) | | 1.00 (0.89 – 1.11) |
| 3 | | 0.93 (0.82 – 1.06) | | 1.11 (0.98 – 1.26) |
| 4 | | 0.83 (0.72 – 0.97) * | | 1.23 (1.06 – 1.42) ** |
| *p-*value for trend | | 0.018 | | 0.002 |
|  | |  | |  |
| **Women** | |  | |  |
| Quartiles | |  | |  |
| 1 | Ref. | | Ref. | |
| 2 | 0.97 (0.78 – 1.19) | | 0.95 (0.78 – 1.17) | |
| 3 | 0.97 (0.73 – 1.30) | | 1.10 (0.83 – 1.47) | |
| 4 | 0.93 (0.64 – 1.35) | | 1.13 (0.78 – 1.62) | |
| *p-*value for trend | 0.828 | | 0.487 | |

Model was adjusted for all factors in model 3 (i.e., baseline age, race, treatment group, diabetes duration, smoking, alcohol, hypertension history, stroke history, and CVD history). Note: CI = confidence interval, DN = diabetic nephropathy, LBMI = lean body mass index, FMI = fat mass index, HR = hazard ratio.

P-value notation: *: p<0.05. **: p<0.01. ***: p<0.001

*Both predicted LBMI and predicted FMI were mutually adjusted for each other

**Supplementary Table 4** Sensitivity analysis of LBMI and FMI in relation to DN by excluding serious adverse event occurring early in the first two years of follow-up among men and women

|  | **HR (95% CI)** | | **HR (95% CI)** |
| --- | --- | --- | --- |
| Men |  | |  |
| Quartiles | Predicted LBMI | | Predicted FMI |
| 1 | Ref. | | Ref. |
| 2 | 0.97 (0.87 – 1.08) | | 1.00 (0.90 – 1.11) |
| 3 | 0.93 (0.82 – 1.06) | | 1.11 (0.98 – 1.26) |
| 4 | 0.83 (0.71 – 0.97) * | | 1.24 (1.07 – 1.43) ** |
| *p-*value for trend | 0.016 | | 0.002 |
|  |  | |  |
| **Women** |  | |  |
| Quartiles |  | |  |
| 1 | | Ref. | Ref. |
| 2 | | 0.98 (0.80 – 1.21) | 0.94 (0.76 – 1.15) |
| 3 | | 0.98 (0.73 – 1.31) | 1.06 (0.81 – 1.43) |
| 4 | | 0.96 (0.66 – 1.39) | 1.09 (0.76 – 1.57) |
| *p-*value for trend | | 0.961 | 0.633 |

Model was adjusted for all factors in model 3 (i.e., baseline age, race, treatment group, diabetes duration, smoking, alcohol, hypertension history, stroke history, and CVD history). Note: CI = confidence interval, DN = diabetic nephropathy, LBMI = lean body mass index, FMI = fat mass index, HR = hazard ratio.

P-value notation: *: p<0.05. **: p<0.01. ***: p<0.001

*Both predicted FMI and predicted LBMI were mutually adjusted for each other

**Supplementary Figure 1** Hazard ratios per 2kg/m^2^–increase in predicted LBMI for incident DN among men.


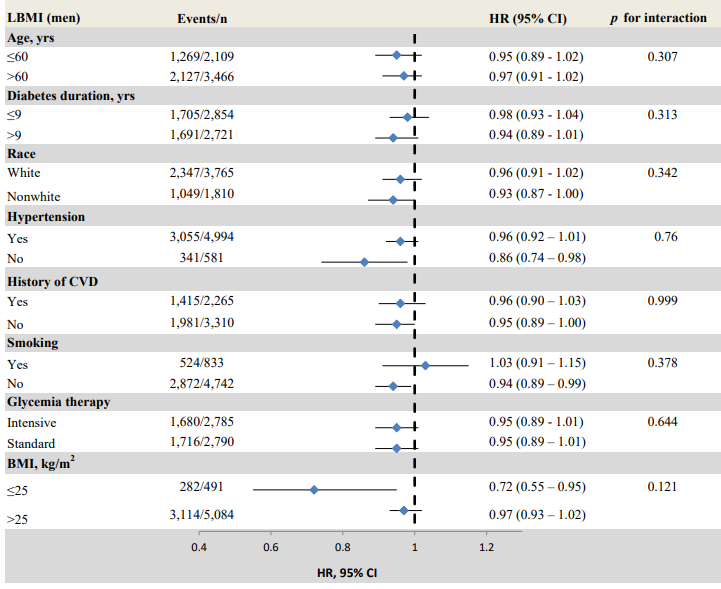


Each stratification was adjusted for FMI and all factors in model 3 (i.e., baseline age, race, treatment group, years of diabetes, smoking, alcohol consumption, hypertension, stroke, and CVD) except for the stratification factor itself. Note: CI = confidence interval, CVD = cardiovascular disease, HR = hazard ratio

**Supplementary Figure 2** Hazard ratios per 2kg/m^2^–increase in predicted FMI for incident DN among men.


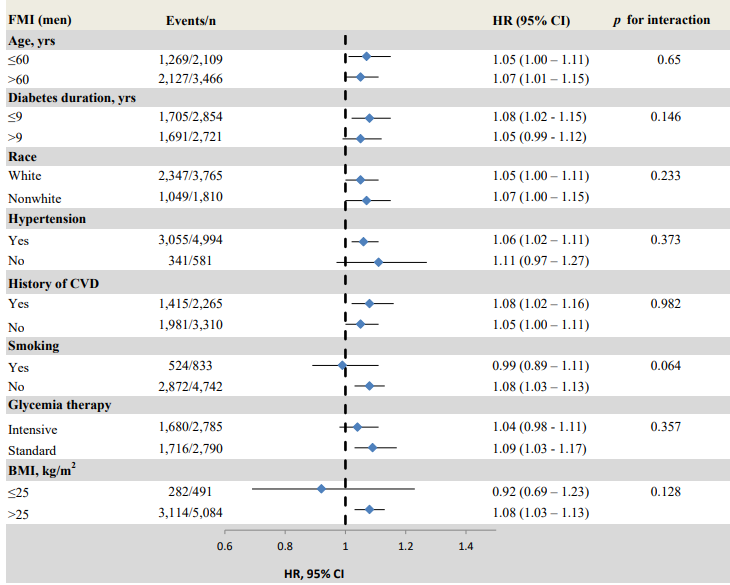


Each stratification was adjusted for LBMI and all factors in model 3 (i.e., baseline age, race, treatment group, years of diabetes, smoking, alcohol consumption, hypertension, stroke, and CVD) except for the stratification factor itself. Note: CI = confidence interval, CVD = cardiovascular disease, HR = hazard ratio

**Supplementary Figure 3** Hazard ratios per 2kg/m^2^–increase in predicted LBMI for incident DN among women.


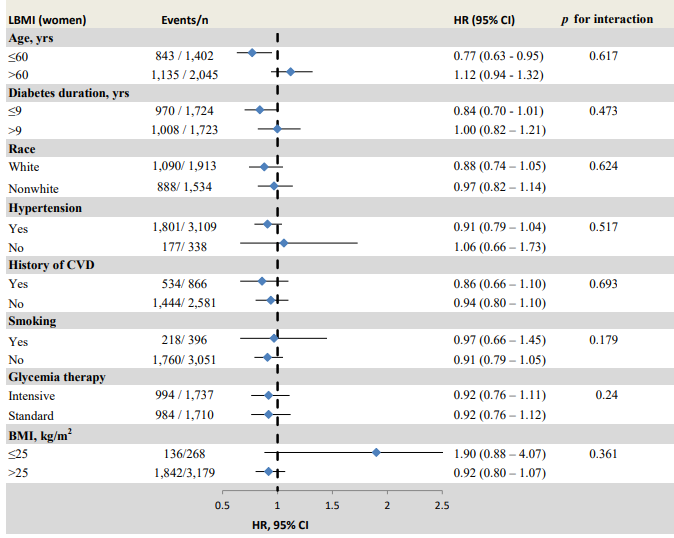


Each stratification was adjusted for FMI and all factors in model 3 (i.e., baseline age, race, treatment group, years of diabetes, smoking, alcohol consumption, hypertension, stroke, and CVD) except for the stratification factor itself. Note: CI = confidence interval, CVD = cardiovascular disease, HR = hazard ratio

**Supplementary Figure 4** Hazard ratios per 2kg/m^2^–increase in predicted FMI for incident DN among women.


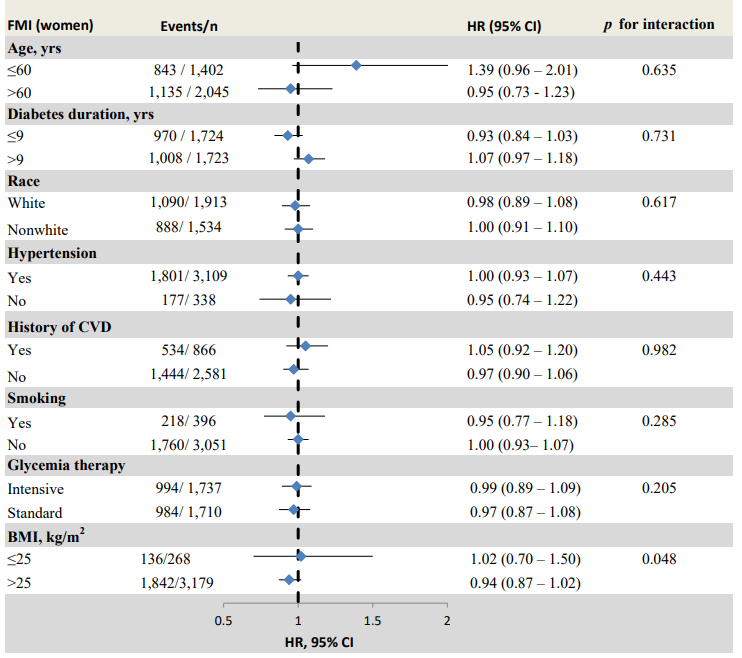


Each stratification was adjusted for LBMI and all factors in model 3 (i.e., baseline age, race, treatment group, years of diabetes, smoking, alcohol consumption, hypertension, stroke, and CVD) except for the stratification factor itself. Note: CI = confidence interval, CVD = cardiovascular disease, HR = hazard ratio
